# ChronoSort: Revealing Hidden Dynamics in AlphaFold3 Structure Predictions

**DOI:** 10.3390/synbio3040018

**Published:** 2025-11-14

**Authors:** Matthew J. Argyle, William P. Heaps, Corbyn Kubalek, Spencer S. Gardiner, Bradley C. Bundy, Dennis Della Corte

**Affiliations:** 1 Department of Physics and Astronomy, Brigham Young University, Provo, UT 84602, USA; 2 Department of Chemical Engineering, Brigham Young University, Provo, UT 84602, USA

**Keywords:** protein engineering, Alphafold3, ensemble, PCA, molecular dynamics

## Abstract

Protein function emerges from dynamic conformational changes, yet structure prediction methods provide only static snapshots. While AlphaFold3 (AF3) predicts protein structures, the potential for extracting dynamic information from its ensemble predictions has remained underexplored. Here, we demonstrate that AF3 structural ensembles contain substantial dynamic information that correlates remarkably well with molecular dynamics simulations (MD). We developed ChronoSort, a novel algorithm that organizes static structure predictions into temporally coherent trajectories by minimizing structural differences between neighboring frames. Through systematic analysis of four diverse protein targets, we show that root-mean-square fluctuations derived from AF3 ensembles can correlate strongly with those from MD (r = 0.53 to 0.84). Principal component analysis reveals that AF3 predictions capture the same collective motion patterns observed in molecular dynamics trajectories, with eigenvector similarities significantly exceeding random distributions. ChronoSort trajectories exhibit structural evolution profiles comparable to MD. These findings suggest that modern AI-based structure prediction tools encode conformational flexibility information that can be systematically extracted without expensive MD. We provide ChronoSort as open-source software to enable broad community adoption. This work offers a novel approach to extracting functional insights from structure prediction tools in minutes, with significant implications for synthetic biology, protein engineering, drug discovery, and structure–function studies.

## 1. Introduction

Protein function is fundamentally linked to structural dynamics, with conformational changes enabling catalysis, binding, and regulatory processes that are essential to biological systems [1–5]. Tools like AlphaFold3 (AF3) provide unprecedented access to high-quality structural models for hundreds of millions of proteins [6–8]. AF3 represents a substantial advancement over previous versions, incorporating a diffusion-based architecture capable of predicting the joint structure of complexes including proteins, nucleic acids, small molecules, ions and modified residues with greatly improved accuracy. However, these predictions fundamentally provide static snapshots of protein structures, while the proteins themselves exist as dynamic ensembles of conformations that fluctuate on timescales ranging from picoseconds to milliseconds [9–11].

The challenge of bridging static structure predictions with dynamic protein behavior has become increasingly prominent [12,13]. Traditional approaches to understanding protein dynamics rely heavily on molecular dynamics simulations (MD), nuclear magnetic resonance spectroscopy (NMR), and other experimental techniques that can probe conformational flexibility [14–17]. While MD provide detailed atomic-level insights into protein motion, they are computationally expensive and require significant expertise to perform and analyze effectively [18,19].

Recently, approaches for predicting the relative populations of protein conformations using AlphaFold2 suggested that AI-powered methods can potentially capture aspects of conformational heterogeneity [20,21]. Additionally, investigations into AlphaFold2’s ability to predict structural ensembles of disordered proteins have shown promise, particularly for regions with low confidence scores [22,23]. A study has even suggested a correlation between AlphaFold2 and MD [24]. However, these studies have primarily focused on highly flexible regions, intrinsically disordered proteins, or only considered one protein. It remains unclear whether AF3 ensemble information from protein structure predictions contain meaningful dynamic signatures.

The confidence scores provided by AlphaFold models, particularly the per-residue predicted Local Distance Difference Test (pLDDT) scores, have been interpreted primarily as measures of structural certainty rather than indicators of dynamic behavior [25,26]. While regions with low confidence scores often correspond to flexible loops or disordered regions, the relationship between confidence patterns and the specific modes of protein motion observed in MD has not been systematically explored [27,28]. This represents a significant gap in our understanding, as extracting dynamic information directly from static predictions could accelerate functional annotation and protein design efforts.

MD can be characterized through principal component analysis (PCA) and normal mode analysis [29–31]. These collective motions, often described by the first few principal components or normal modes, frequently correspond to functionally relevant conformational changes [32,33]. The question of whether similar collective motion patterns might be encoded within the structural variations in AF3 ensemble predictions has remained largely unexplored, despite its potential significance for structure-based drug design and protein engineering applications [34–37].

Furthermore, the temporal organization of protein conformations—how structures transition between different states—represents a critical aspect of protein dynamics that traditional static predictions do not capture [38,39]. Methods for organizing structural ensembles into meaningful temporal sequences could provide insights into folding pathways, conformational transitions, and functional mechanisms [40,41]. The development of such approaches would represent a significant advancement in our ability to understand protein dynamics from computational predictions alone.

Here, we present a case study investigating the dynamic information content of AF3 ensemble predictions. We introduce ChronoSort, a novel computational approach that organizes static structural predictions into temporal trajectories by minimizing structural differences between neighboring frames, effectively creating pseudo-molecular dynamics trajectories from ensemble predictions. Through systematic comparison with explicit MD across diverse protein targets, we demonstrate that AF3 ensemble predictions contain dynamic information that correlates strongly with experimentally derived and simulation-based measures of protein flexibility.

## 2. Results and Discussion

### 2.1. Conceptual Framework: Extracting Dynamics from Static Predictions

**Figure 1.**
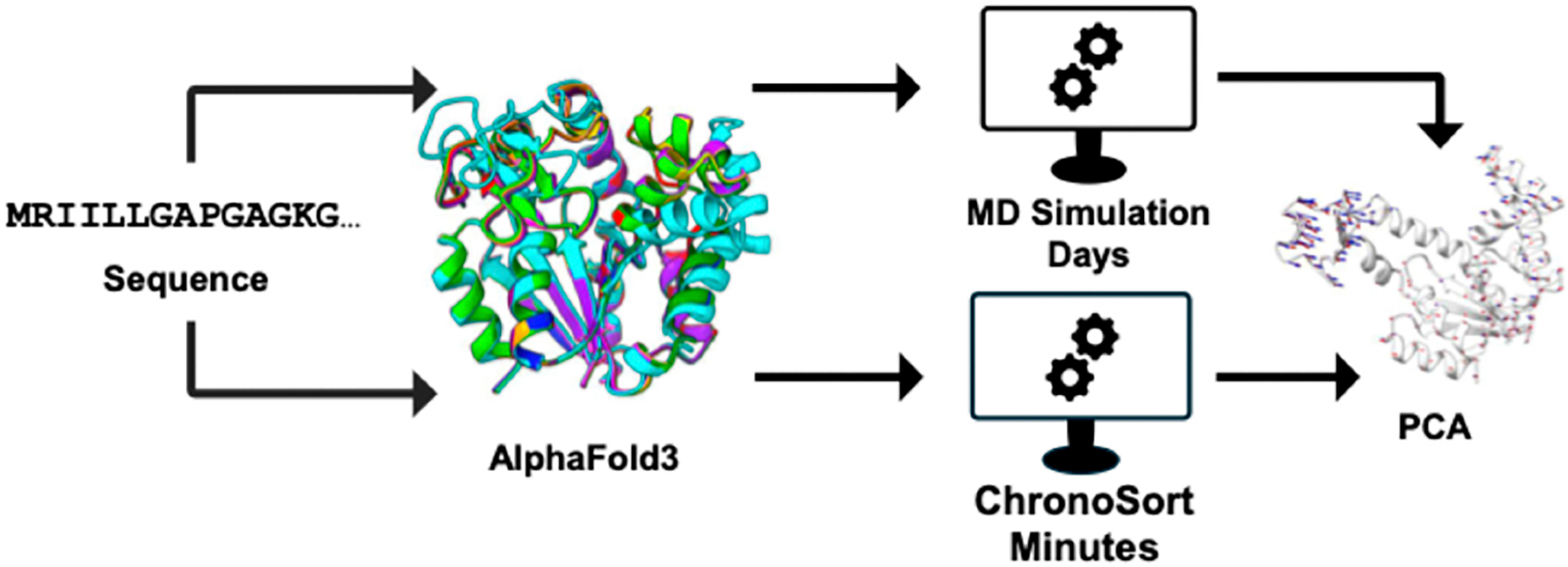
Graphical abstract illustrating the ChronoSort methodology. AF3 ensemble predictions (**left**) are processed through the ChronoSort algorithm to generate temporally ordered trajectories (**bottom center**) for rapid principal component analysis (PCA, (**right**)) and root-mean-square fluctuation (RMSF) which could speed up design–build–test–learn cycles by circumventing costly and time-consuming MD analysis (**top center**).

The central premise of this work is that AF3 prediction ensembles contain latent dynamic information that can be systematically extracted and compared to traditional MD. This concept represents a paradigm shift from viewing structure prediction tools as sources of static snapshots to recognizing them as repositories of conformational heterogeneity that reflect the underlying dynamics of protein systems (Figure 1).

Our approach leverages the observation that modern deep learning structure prediction models, while not explicitly trained on dynamic data, encode information about conformational flexibility through their sampling of sequence–structure relationships during training. Recent studies have found that useful information about protein dynamics can be extracted from the confidence scores of AlphaFold2 structural outputs for a variety of protein targets [42–45]. While some intrinsically disordered proteins have been investigated prior, the systematic extraction and validation of dynamic information from AF3 ensembles has not been thoroughly explored, particularly through direct comparison with MD [46].

### 2.2. Initial Discovery: Dynamic Signatures in NanoLuc Luciferase

**Figure 2.**
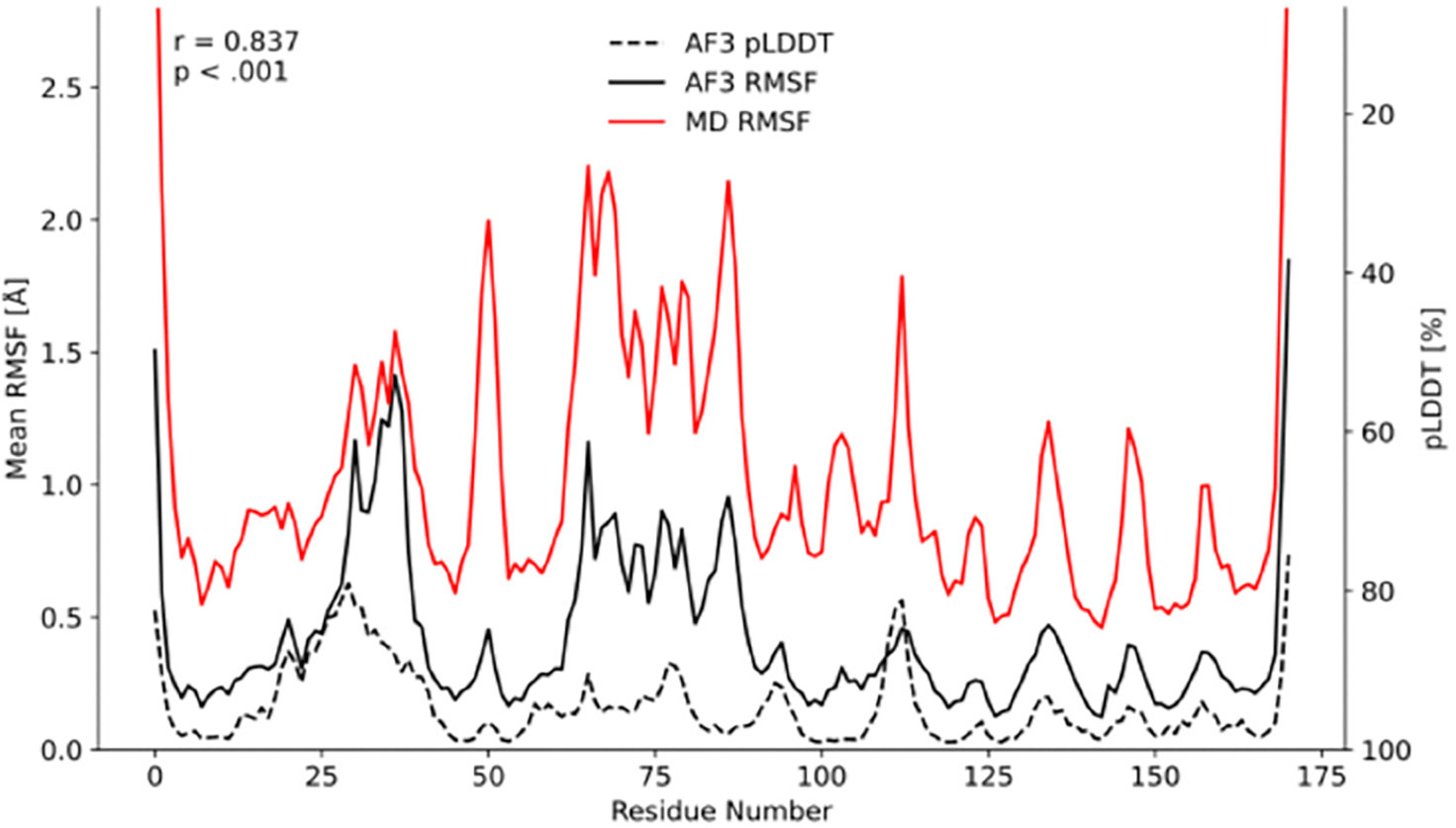
Root-mean-square fluctuation (RMSF) comparison for NanoLuc luciferase. Per-residue RMSF values calculated from MD (red line, averaged over three 250 ns replicates) show strong correlation with RMSF derived from AF3 structural ensemble (black line, *n* = 100 structures). The correlation coefficient (r = 0.82, *p* < 0.001) indicates that AF3 predictions capture the major flexibility patterns observed in explicit molecular dynamics simulations.

Our investigation began with the observation of strong correlations between AF3 structural variations and MD-derived flexibility patterns in NanoLuc luciferase, a 19.1 kDa enzyme that we were studying for protein engineering applications [47]. The correlation between AF3-derived and MD-derived RMSF was remarkably high (r = 0.84), suggesting that the structural ensemble predictions contained meaningful dynamic information (Figure 2).

This initial finding was particularly striking because AF3 was neither designed nor trained to predict protein dynamics, yet the structural variations in its predictions appeared to encode information about conformational flexibility. It is also noteworthy that pLDDT does not correlate as strongly with MD as the AF3 RMSF. The strong correlation observed in NanoLuc prompted us to investigate whether this phenomenon was generalizable across different protein families and structural motifs.

### 2.3. Systematic Validation Across Diverse Protein Targets

**Figure 3.**
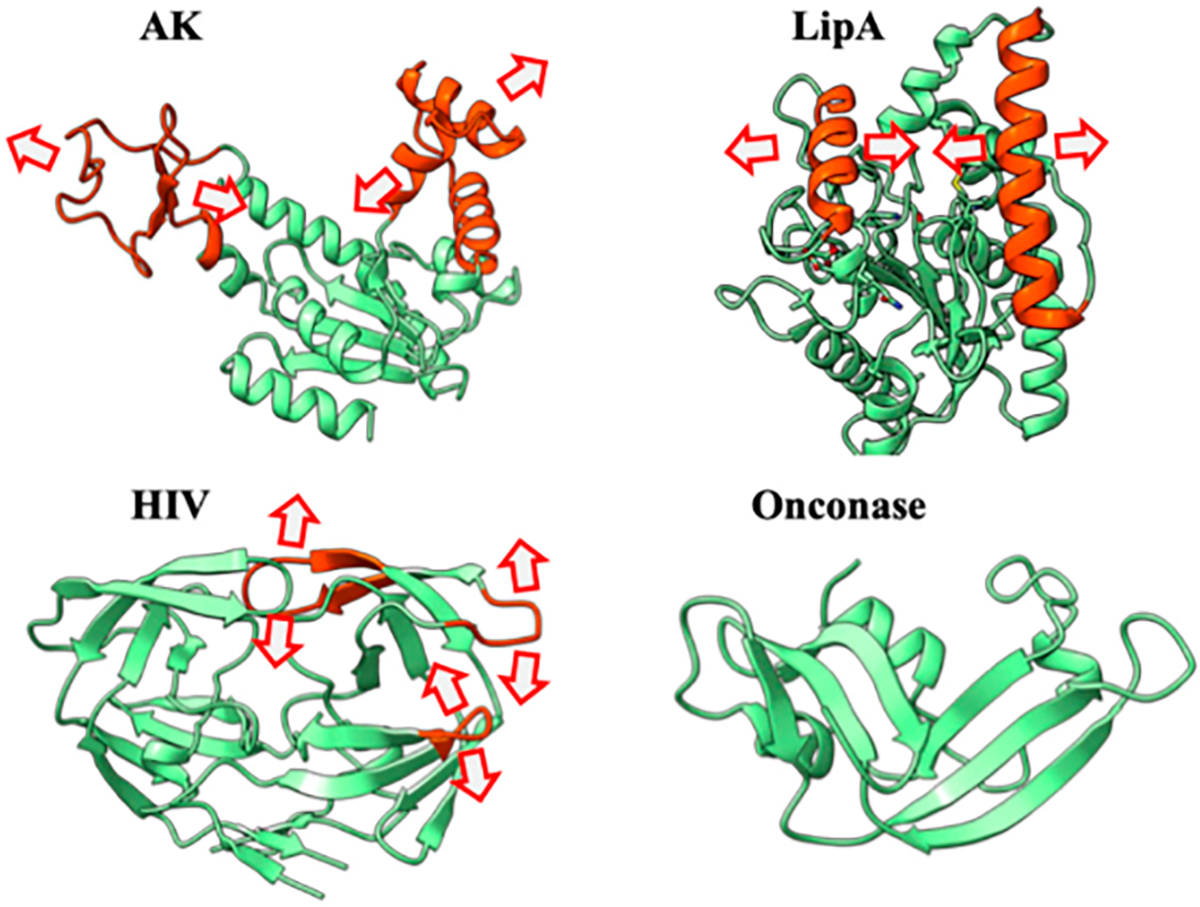
Structural overview of protein targets and their predominant motions. 2 × 2 panel showing the four protein targets: (**Upper left**) Adenylate kinase (PDB: 4AKE) with characteristic domain closure motion; (**Upper right**) Bacterial subtilis lipase A (PDB: 1EX9) with lid domain motion; (**Lower left**) HIV-1 protease (PDB: 1HPV) highlighting flap dynamics; (**Lower right**) Onconase (PDB: 1ONC) with loop flexibility patterns. Red arrows indicate the primary axes of motion identified in previous protein dynamics studies.

To assess the generalizability of our findings, we selected four additional protein targets representing diverse structural families and functional classes: bacterial lipase A, adenylate kinase, HIV-1 protease, and Onconase (Figure 3). These proteins span different fold families and exhibit distinct types of functional motions, from lid movements in lipases to domain closure in kinases and flap dynamics in proteases.

The selection of these targets was strategic, as each represents well-characterized systems with established functional motions that have been extensively studied through both experimental and computational approaches.

### 2.4. ChronoSort Algorithm Validates Temporal Organization

**Figure 4.**
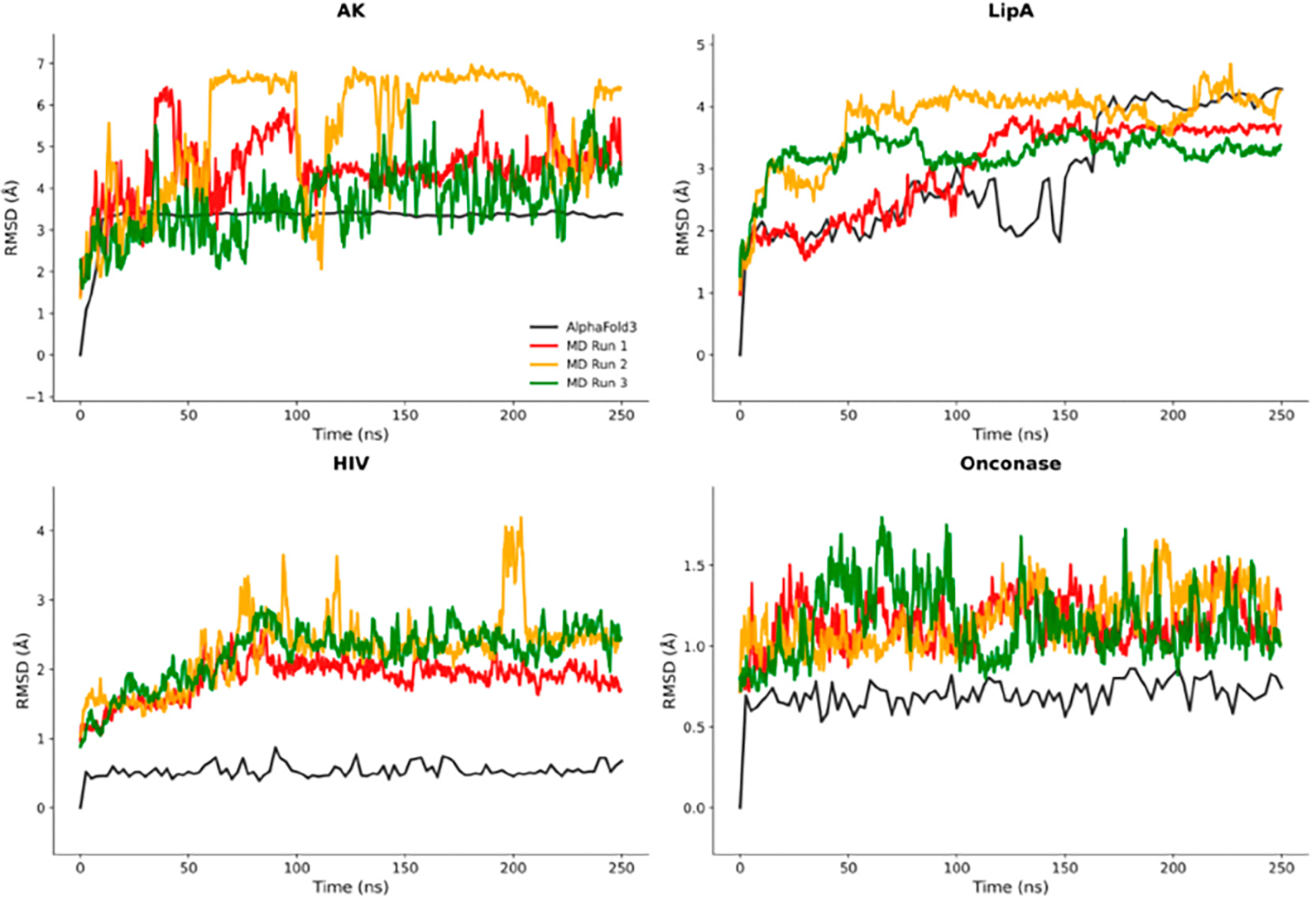
RMSD analysis of ChronoSort trajectories compared to molecular dynamics simulations. 2 × 2 panel showing RMSD evolution for all four protein targets: (**Upper left**) Adenylate Kinase, (**Upper right**) Bacterial subtilis lipase A, (**Lower left**) HIV-1 protease, (**Lower right**) Onconase. For each system, molecular dynamics trajectories (three replicates, colored lines) are compared with the ChronoSort trajectory derived from AF3 ensemble predictions (black line).

The ChronoSort algorithm successfully organized AF3 ensemble predictions into temporally coherent trajectories that exhibited pairwise Cα root-mean-square deviation (RMSD) profiles similar to those observed in MDs (Figure 4).

The RMSD profiles generated by ChronoSort indicate several important characteristics that validate the biological relevance of the extracted temporal organization. As the RMSD magnitudes of all proteins except HIV-1 protease are comparable, ChronoSort appears to sample similar spreads of conformational distributions as those seen in MD.

### 2.5. Quantitative Validation of Flexibility Patterns

**Figure 5.**
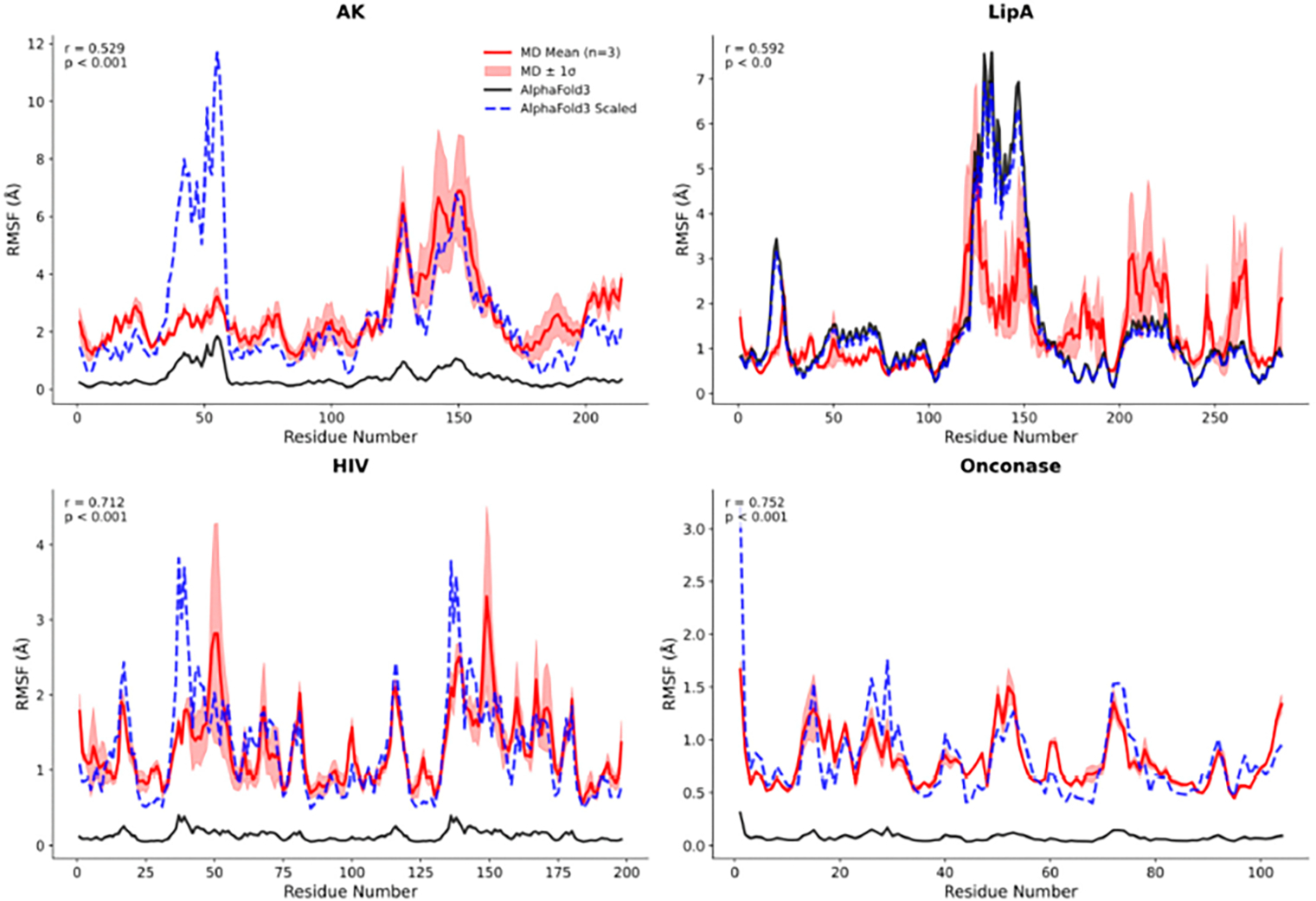
Root-mean-square fluctuation correlations across protein targets. 2 × 2 panel comparing MD-derived RMSF (purple lines, averaged over three replicates) with AF3-derived RMSF (black lines) for: (**Upper left**) Adenylate Kinase (r = 0.53), (**Upper right**) Bacterial subtilis lipase A (r = 0.59), (**Lower left**) HIV-1 protease (r = 0.71), (**Lower right**) Onconase (r = 0.75). Shaded regions indicate standard deviation across MD replicates. The dashed blue lines represent AF3 RMSF values scaled to same area under the curve as MD RMSF.

Additional evidence for the biological relevance of dynamic information in AF3 ensembles came from systematic comparison of RMSF patterns. Across all four protein targets, we observed consistently high correlations between AF3-derived and MD-derived RMSF values, with correlation coefficients (r) ranging from 0.53 to 0.75 (Figure 5).

The fact that flexible regions identified by AF3 generally show consistent agreement with MD-identified regions suggests that the static predictions are capturing fundamental aspects of protein dynamics that are robust across proteins with different dynamics.

The biological significance of these motions is further supported by the correlations between the peaks and the valleys, and their consistency with known functional elements. For example, the highest RMSF and second highest AF3 RMSF values in adenylate kinase correspond to the lid domain that undergoes large-scale conformational changes during the catalytic cycle, while in HIV-1 protease the peak flexibility occurs in the flap regions that are critical for substrate binding and product release.

### 2.6. Analysis of Side Chain Conformation

**Figure 6.**
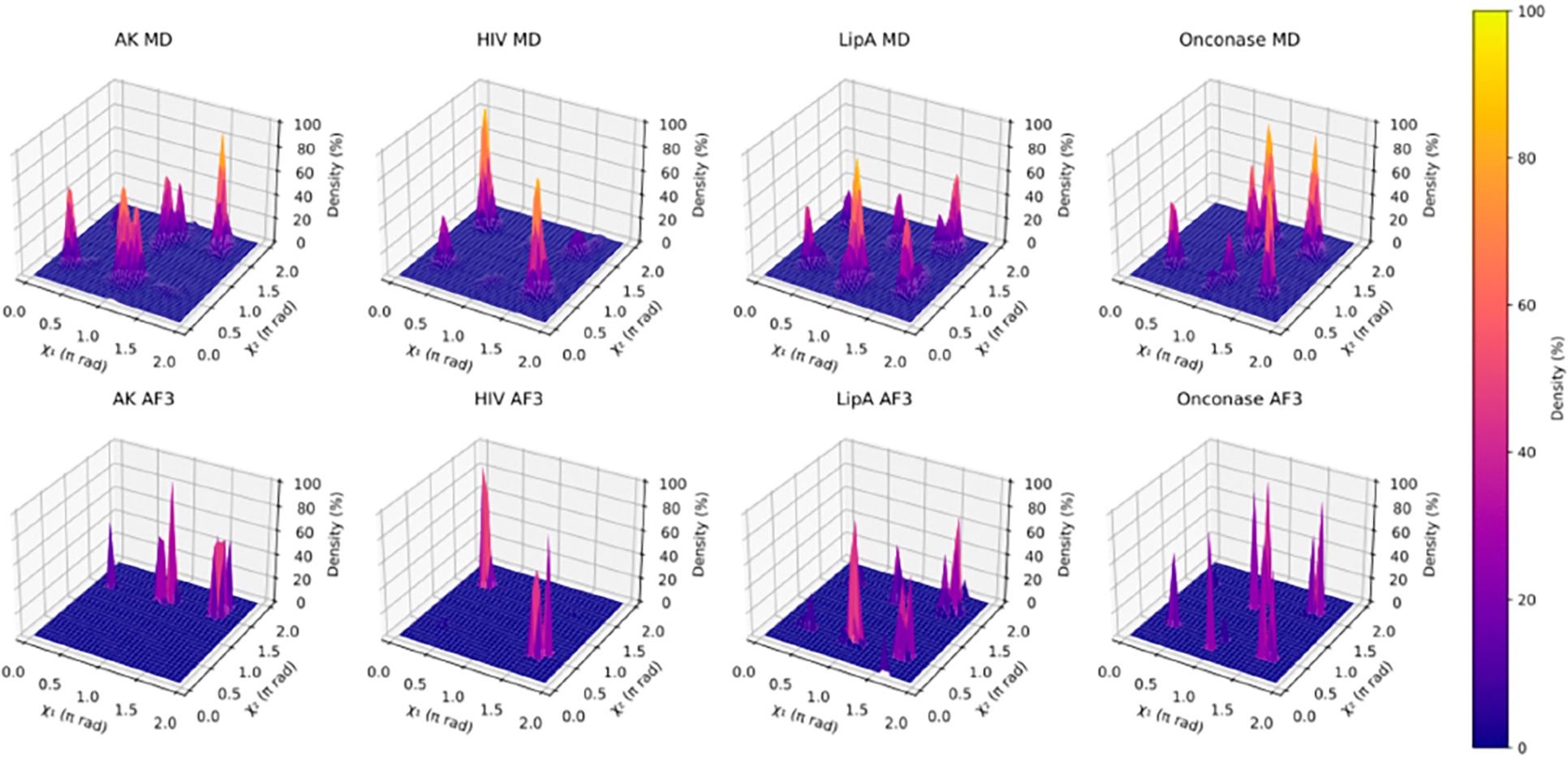
Side chain dihedral χ^1^ angles plotted against χ^2^ angles for the aromatic residues PHE, TRP, and TYR with the prevalence of occurrence on the *z*-axis. MD values are on the left and AF3 are on the right. The substantially larger number of frames in the MD trajectory led to a smoother plot.

Distribution of side chain conformation of aromatic residues (phenylalanine, tryptophan, and tyrosine) from AF3 ensembles and MD simulations were also considered (Figure 6). Since AF3 predicts viable protein structures, it was expected that side chain torsion angles χ^1^ and χ^2^ would be more restricted to those reported in rotamer libraries while the MD simulation would contain transition states between them.

We find that AF3 ensembles sample a subset of the side chain conformations observed in MD, with the notable exception of AF3 not predicting the secondary χ^2^ conformation for adenylate kinase. This is not surprising, as AF3 was expected to reproduce torsion distributions found in rotamer libraries and not dynamic intermediates.

### 2.7. Principal Component Analysis Reveals Shared Motion Patterns

**Figure 7.**
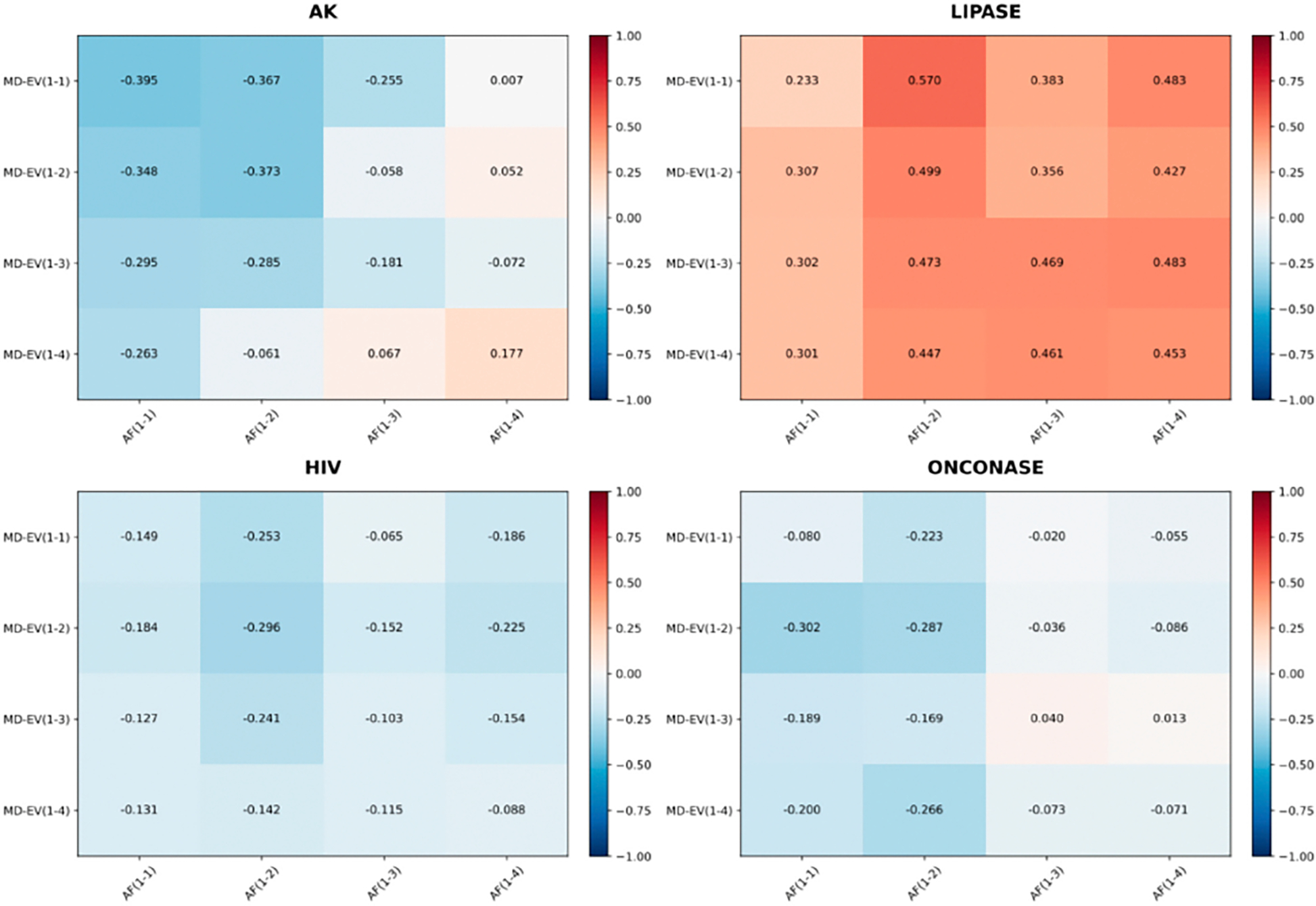
Eigenvector similarity analysis across protein targets. Cosine similarity matrices comparing principal component eigenvectors derived from molecular dynamics simulations (MD-EV(1-x) representing the first x eigenvectors added together and AF(1-x) is similar). Color scale indicates cosine similarity values from −1 (opposite) to 1 (identical).

To investigate whether AF3 ensembles capture not only the magnitude of flexibility but also the directional patterns of collective motion, we performed PCA on both MD trajectories and AF3 structural ensembles. The results revealed striking similarities in the eigenvector patterns, with cosine similarity values significantly higher than those expected from random vectors of equivalent dimensionality for all proteins tested (Figure 7). The odds of randomly obtaining vectors with equal or greater similarity are approximately 1 in 1.8 × 10^25^ for adenylate kinase, 1 in 6.6 × 10^75^ for lipase A, 1 in 1.6 × 10^13^ for HIV-1 protease, and 1 in 5.0 × 10^8^ for Onconase.

This finding has important implications for drug discovery applications where PCA is used to understand protein–ligand interactions and conformational changes. The ability to extract reliable information about collective motions directly from structure predictions, which takes only minutes, could significantly accelerate the identification of druggable conformational states and allosteric binding sites. Naturally, only magnitudes of cosine similarities are of importance as projections along anti-parallel vectors result in the same motion as projections along parallel vectors.

### 2.8. Visual Validation Through Porcupine Plot Analysis

**Figure 8.**
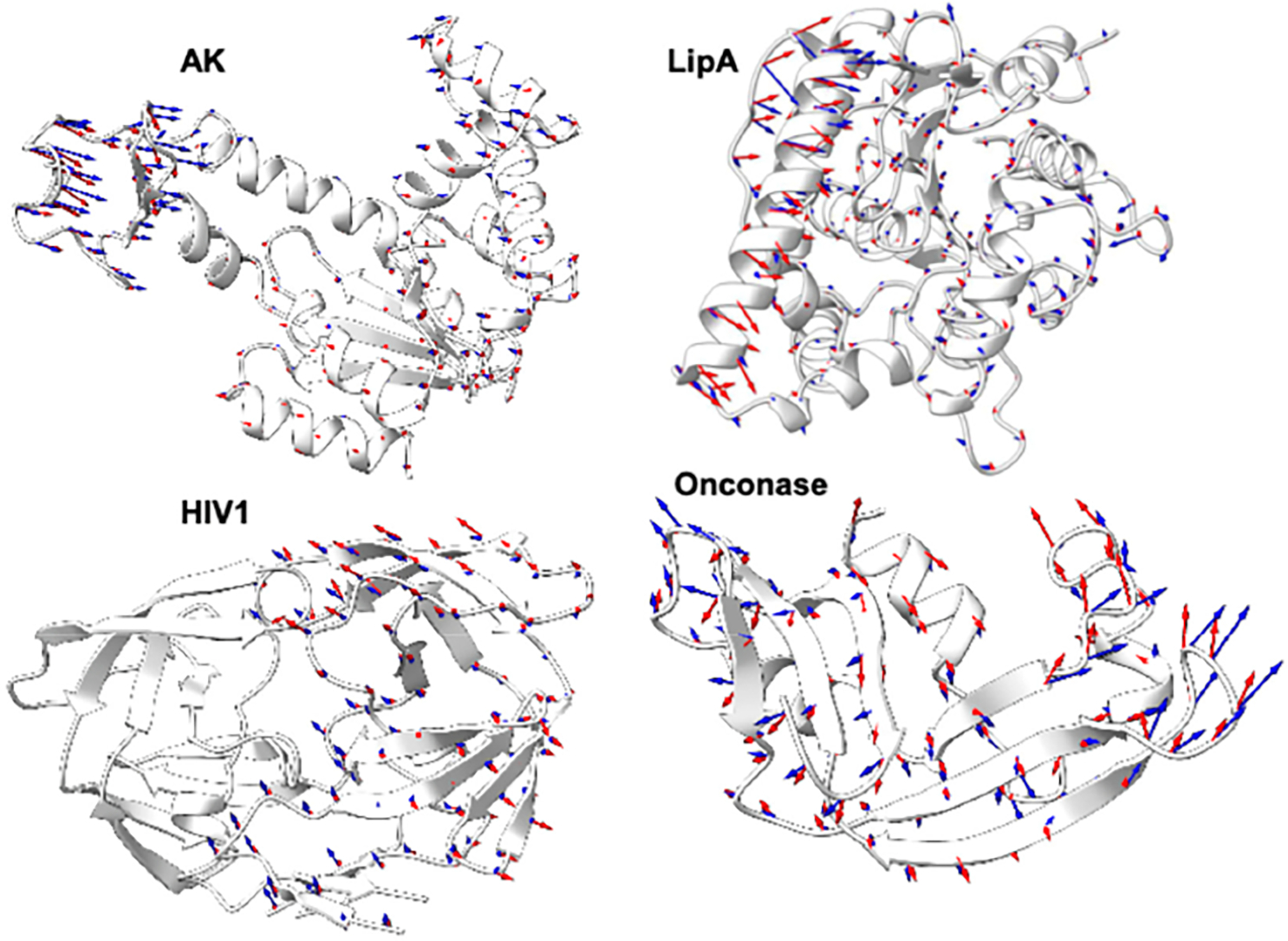
Porcupine plot comparison of principal motion patterns. 2 × 2 panel showing the first two eigenvectors added together for each protein target derived from MD (blue vectors) and AF3 ensembles (red vectors): (**Upper left**) Adenylate Kinase showing lid domain motion, (**Upper right**) Bacterial subtilis lipase A displaying domain closure, (**Lower left**) HIV-1 protease highlighting flap dynamics, (**Lower right**) Onconase showing loop flexibility. The eigenvectors with negative cosine similarity were negated for visualization.

Porcupine plots display the direction and magnitude of atomic displacements along each principal component. The visual similarity between the first two principal components from MD-derived and AF3-derived motion patterns (Figure 8) reveals nearly identical directional preferences and relative magnitudes across all four protein targets. This indicates that the conformational changes predicted by AF3 are similar to those extracted from MD.

The porcupine plots show that AF3 ensembles correctly identify the functional motion patterns that are characteristic of each protein family. In lipase A, both approaches identified the opening and closing motion of the lid domain that controls substrate access to the active site. For adenylate kinase, the dominant motion corresponded to the well-characterized domain closure that brings the ATP and AMP binding sites together during the catalytic cycle. In HIV-1 protease, the primary motion involved the flap regions that gate substrate binding. In Onconase, the dominant patterns reflected the flexibility of surface loops involved in RNA binding. It is also significant that AF3 ensembles can correctly predict the dynamics of Onconase since AF3 predicts Onconase’s structure with extreme levels of certainty. This suggests that this latent dynamical information in the ensemble is not a result of sampling from AF3’s uncertainty.

### 2.9. Implications for Structure–Function Relationships

The strong correlations observed across multiple metrics and protein systems suggest that AF3 ensemble predictions contain a wealth of dynamic information that has been largely overlooked in structure–function analyses. Recent work has begun to integrate AF3 confidence scores into enhanced protein flexibility simulations [48], but our results indicate that the full structural ensembles provide far richer dynamic information than confidence scores alone.

The traditional paradigm of using single static structures to infer function is increasingly recognized as insufficient, particularly for understanding allosteric regulation, conformational selection mechanisms, and the effects of mutations on protein dynamics. Our results suggest that much of this missing dynamic information can be extracted directly from structure prediction ensembles, which greatly accelerates the speed at which dynamic information can be generated.

The ability to rapidly assess protein dynamics from structure predictions could speed up several areas of synthetic biological research. In protein engineering, understanding flexibility patterns is crucial for designing mutations that preserve or modify specific functional motions [49]. In drug discovery, knowledge of conformational dynamics is essential for identifying allosteric binding sites and understanding binding mechanisms [50]. In structural biology, dynamic information helps interpret the functional significance of structural features and guides experimental design [12].

### 2.10. Methodological Considerations and Limitations

While our results are highly encouraging, several important limitations must be acknowledged. First, our analysis focused on relatively small, well-folded proteins, and the generalizability to larger, multidomain proteins or membrane proteins remains to be established. Recent work suggests that AlphaFold-Multimer can capture dynamics of intrinsically disordered regions, but systematic validation across diverse protein classes is needed [51].

Second, the ChronoSort algorithm, while effective at organizing structural ensembles into meaningful trajectories, represents only one possible approach to temporal organization. Alternative methods based on transition state theory or Markov state modeling might provide additional insights into the conformational transitions encoded in static predictions [52]. The current implementation also assumes that the ensemble predictions sample conformations along realistic transition pathways, which may not always be the case.

Third, because AF3 has no concept of temperature, pH, or solution environment, ChronoSort will not be able to predict the effects of these factors on protein dynamics. This highlights that ChronoSort is not a tool to replace MD but to aid it as a method to generate dynamic information more rapidly.

Fourth, our analysis was limited to backbone dynamics, while many functional motions involve side chain rearrangements that may not be well-captured in Cα-based analyses [53]. Future extensions of this work should incorporate all-atom analyses and validation against experimental measures of side chain dynamics, such as NMR relaxation data although most of the proteins studied are larger than what NMR can traditionally resolve.

Overall, the ChronoSort method using AF3 provides a rapid estimate of the Cα-based RMSF and PCA profiles which could greatly speed up design–build–test–learn cycles of protein engineering, drug discovery, and structure–function studies. This technology is particularly useful to experimentalists with limited MD training, as the learning curve for using AF3 is minimal for the end user compared to MD. It is also useful to those without large computational resources as the AF3 server can be used to generate ensembles.

## 3. Methods

### 3.1. Protein Target Preparation

Four diverse protein targets were selected to represent different structural families and functional classes: NanoLuc luciferase (PDB: 5IBO), bacterial lipase A from *Bacillus subtilis* (PDB: 1EX9), adenylate kinase from *Escherichia coli* (PDB: 4AKE), HIV-1 protease (PDB: 1HPV), and Onconase from *Rana pipiens* (PDB: 1ONC) [54–58]. Amino acid sequences were retrieved directly from the Protein Data Bank entries, with signal peptides and non-canonical residues removed where appropriate to ensure compatibility with both AF3 prediction protocols and MD force fields.

### 3.2. AlphaFold3 Ensemble Generation

AF3 predictions were generated using a local installation of AF3 with weights kindly provided by DeepMind [7]. To create structural ensembles, 100 independent predictions were generated for each target protein using 20 different seeds, each seed generating 5 unique models, following established protocols for ensemble generation from deep learning structure prediction models. 100 models were used to decrease the susceptibility to the random variance of AF3 structure predictions and create ensembles of consistently similar PCA across the tested proteins. Each prediction was carried out with identical input sequences but unique initialization states to sample the conformational heterogeneity encoded in the AF3 model. All predicted structures were downloaded in PDB format and subjected to quality control checks to ensure proper chain assignment and coordinate integrity.

### 3.3. Molecular Dynamics Simulations

All MDs were performed using GROMACS 2022.4 with the AMBER03 force field [59]. Initial structures were prepared by taking the first model from each target’s crystallographic structure, with missing hydrogen atoms added using the pdb2gmx tool. Each protein was solvated in a rectangular box with TIP3P water molecules, maintaining a minimum distance of 1.0 nm between the protein and box boundaries. The systems were neutralized by adding appropriate counter-ions (Na^+^ and Cl^−^) to achieve physiological ionic strength (0.15 M NaCl).

Energy minimization was performed using the steepest descent algorithm until the maximum force fell below 1000 kJ mol^−1^ nm^−1^. Subsequently, the systems underwent NVT equilibration at 300 K for 100 ps using the V-rescale thermostat, followed by NPT equilibration at 300 K and 1 bar for 100 ps using the Parrinello–Rahman barostat. Production runs were conducted for 250 ns in triplicate for each target, with coordinates saved every 10 ps, resulting in 25,000 frames per trajectory. All bond lengths involving hydrogen atoms were constrained using the LINCS algorithm, allowing for a 2 fs integration time step.

### 3.4. ChronoSort Algorithm

The ChronoSort algorithm was developed to organize AF3 ensemble predictions into temporally coherent trajectories by minimizing RMSD between consecutive frames. The algorithm takes a folder of uniquely named structures as input. First, pairwise Cα RMSD values were calculated between all predicted structures in the ensemble after optimal superposition using the Kabsch algorithm [60]. This generates a symmetric n × n distance matrix D, where D[i,j] represents the RMSD between structures i and j.

The optimal temporal ordering was determined by solving a variant of the traveling salesman problem. ChronoSort finds the path through the distance matrix that minimizes the total RMSD between consecutive structures. The algorithm employs a greedy nearest-neighbor heuristic. For this, it starts from each possible starting structure, selects the next closest structure in RMSD—for structure i the minimum in D[i, :] row that is not in the path—and continuous so until all structures are connected through a path. This yields n possible paths, of which the one with the smallest total RMSD is used to compile structures into a multi-model structure file to generate the ChronoSort trajectory. This trajectory can then be analyzed using protocols developed for MD.

### 3.5. Root-Mean-Squared Deviation Analysis

RMSD calculations were performed on Cα atoms after least-squares superposition to the initial frame of each trajectory. For MD trajectories, RMSD was calculated as a function of simulation time. For ChronoSort trajectories, frames were assigned artificial time points by dividing the 250 ns simulation time window by 100 frames, yielding a temporal resolution of 2.5 ns per frame to enable direct comparison with MD trajectories. RMSD analysis was performed using GROMACS analysis tools, with results visualized using matplotlib and compared across the three MD replicates and the single ChronoSort trajectory for each target.

### 3.6. Root-Mean-Square Fluctuation Calculations

RMSF values were calculated for Cα atoms after center-of-mass alignment of all trajectory frames. For MD, RMSF was computed using the standard definition [61]. For AF3 ensembles, RMSF was calculated as the root-mean-square distance of each Cα atom from its mean predicted position across the ensemble. RMSF values were computed using GROMACS gmx rmsf tool for MD trajectories and custom Python scripts for AF3 ensembles, with results compared using Pearson correlation coefficients.

### 3.7. Side Chain Analysis

Side chain analysis was performed using 3D Janin plots from MDAnalysis 2.9.0. For the aromatic residues phenylalanine, tryptophan, and tyrosine, the χ^1^ and χ^2^ dihedral angles were extracted from the MD trajectories and converted to radians. These angles were plotted against each other, with point height representing relative density.

### 3.8. Principal Component Analysis

Principal component analysis was performed on Cα coordinates after removal of center of mass translational and rotational motions. Covariance matrices were constructed from the coordinate fluctuations, and eigenvalue decomposition was performed to obtain principal components (eigenvectors) and their associated eigenvalues. The number of eigenvectors retained for analysis was determined by the cumulative eigenvalue criterion, typically encompassing 80% of the total variance, corresponding to approximately the first 4 eigenvectors for most systems.

Cosine similarity between eigenvectors was calculated using the dot product of normalized vectors. For validation, pairwise cosine similarities were first computed between eigenvectors from independent MD replicates to establish the baseline reproducibility of principal modes. Subsequently, cosine similarities were calculated between MD-derived and AF3-derived eigenvectors. Statistical significance was assessed by comparing observed similarities to null distributions generated from randomly oriented vectors of equivalent dimensionality.

### 3.9. Eigenvector Visualization

Principal component eigenvectors were visualized using porcupine plots. These plots display the protein backbone as a cartoon representation with vectors emanating from each Cα position, indicating the direction and magnitude of atomic displacements along each principal component. The most similar eigenvector pairs between MD and AF3 analyses, as determined by cosine similarity calculations, were selected for comparative visualization. Porcupine plots were generated using ChimeraX 1.9 with custom scripts to overlay MD and AF3 eigenvectors in different colors, allowing direct visual comparison of the dominant motion patterns captured by each approach [62].

### 3.10. Eigenvector Similarity Analysis

The probability of observing a vector within a specified angular distance from a reference vector in *n*-dimensional space can be expressed as the ratio of the surface area of a hyperspherical cap to the total surface area of the hypersphere. For a unit *n*-sphere, the fraction of the surface area contained within a polar angle cos(θ) from a fixed axis is given by the complemented regularized incomplete beta function:

$$P\left(\Theta \leq \cos\left(\theta \right)\right)=I_{\left(1+\theta \right)/2}^{c}(\frac{n\;-\;1}{2},\;\frac{n\;-\;1}{2}),$$

where Θ is the angle between a random vector and the reference vector and $I_{z}^{c}(a,\;b)$ is the complementary regularized incomplete beta function. This expression gives the probability that a uniformly distributed random point on the surface of the unit n-sphere lies at an angle greater than or equal to arccos(θ) from a given reference direction [63].

### 3.11. Statistical Analysis

All correlation analyses were performed using Pearson correlation coefficients, with significance assessed using two-tailed t-tests. All statistical analyses were performed using SciPy and NumPy in Python 3.12 [64,65].

## Data Availability

All data and methods used in this paper can be found on the ChronSort GitHub. https://github.com/dellacortelab/chronosort accessed on 6 November 2025.

## Abbreviations

AF3: AlphaFold3
MD: Molecular dynamics simulations
NMR: Nuclear magnetic resonance spectroscopy
PCA: Principal component analysis
RMSF: Root-mean-squared fluctuation
RMSD: Root-mean-squared deviation
pLDDT: per-residue predicted Local Distance Difference Test
